# Dogs and wolves differ in their response allocation to their owner/caregiver or food in a concurrent choice procedure

**DOI:** 10.7717/peerj.12834

**Published:** 2022-02-15

**Authors:** Lindsay Isernia, Clive D.L. Wynne, Leanna House, Erica N. Feuerbacher

**Affiliations:** 1 Department of Animal and Poultry Sciences, Virginia Polytechnic Institute and State University (Virginia Tech), Blacksburg, VA, United States of America; 2 Department of Psychology, Arizona State University, Tempe, AZ, United States of America; 3 Department of Statistics, Virginia Polytechnic Institute and State University (Virginia Tech), Blacksburg, VA, United States of America

**Keywords:** Domestic dog, Wolf, Owner, Food, Preference

## Abstract

Dogs and wolves both show attachment-like behaviors to their owners/caregivers, including exploring more in the presence of the owner/caregiver, and greeting the owner/caregiver more effusively after an absence. Concurrent choice studies can elucidate dogs’ and wolves’ relationship to their owners/caregivers by assessing their preference for the owner/caregiver compared to other stimuli. While previous research has used concurrent choice paradigms to evaluate dogs’ and wolves’ preference between humans giving social interaction or humans giving food, no research has explored their preferences for an owner/caregiver compared to food when the food is not delivered by a human. In the current study, we investigated whether dogs and hand-reared wolves preferred their owner/caregiver or food, unassociated with a human, when they had been equally deprived of each stimulus (at least 4 hours). Each canid experienced four trials; we measured first choice and time spent with each alternative. Dogs overall did not show a preference for the owner or food. Wolves, on the other hand, tended to show a preference for food in both measures. We observed a range of individual variation in both measures, although dogs showed more individual variation. The differences we observed between dogs and wolves align with prior research comparing wolf and dog behavior directed towards humans; however, the reasons for this differential responding could be due to a variety of factors beyond phylogeny.

## Introduction

The relationship dogs have with humans has received much attention in the past two decades, starting with investigations into dogs’ problem-solving abilities and how those correlated with the relationship dogs had with their owners (Topál, Miklósi & Csányi, 1997). Similar research has been conducted with wolves and their interactions with caregivers and other humans (*e.g.*, Udell, Dorey & Wynne, 2008; Wirobski et al., 2021). One way of assessing dogs’ and wolves’ relationship with owners/caregivers is whether they show differential responding to owners/caregivers compared to strangers. Across multiple measures, in a variety of testing paradigms, dogs show clear differences in their behavior towards their owner when compared to their behavior towards strangers. In strange situation tests, modified from infant-parent attachment tests (Ainsworth & Bell, 1970), dogs show attachment-like behaviors to their owners similar to those of infants to their parents (Topál et al., 1998). Researchers found that owners functioned as a secure base for the dog (Palmer & Custance, 2008; Horn, Huber & Range, 2013); that is, when owners were present dogs were more likely to explore and interact with objects in the room than if the dog was with a stranger (c.f., Hansen Wheat, Larsson & Temrin, 2020: dogs explored as much in the presence of their owner as a stranger). Additionally, after separation, dogs showed more proximity-seeking behaviors, and more enthusiastic and longer-lasting greetings, when the owner returned compared to a stranger (Prato-Previde et al., 2003).

Other experimental paradigms have also found that dogs differentially respond to owners compared to others, showing a range of prosocial behaviors more frequently to the owner. For example, dogs gazed longer at their owners than strangers during separation, in aversive situations, and during play (Kerepesi, Dóka & Miklósi, 2015); as well as when the owner and stranger both entered a room, walked across the room, and exited from the room Mongillo et al. (2010). Mongillo et al. (2010) also found that dogs preferentially attended to locations where they last saw their owner compared to the location where they last saw a stranger. Similarly, when petted by the owner compared to a stranger, dogs showed more approach behaviors and more appeasement behaviors (Kuhne, Hößler & Struwe, 2012). Finally, in a concurrent choice between an owner and a stranger, each of whom provided petting, we found that dogs spent significantly more time with their owner than the stranger in an unfamiliar setting (Feuerbacher & Wynne, 2017), paralleling the results from the strange situation test (*e.g.*, Palmer & Custance, 2008; Horn, Huber & Range, 2013). Nevertheless, in a familiar environment (the dog’s home), the dog spent more time with the stranger than the owner, which points to dogs being generally sociable with humans (vonHoldt et al., 2017; Lazzaroni et al., 2020), even as they show differential responding to their owners.

The attachment-like behaviors observed in dogs, in fact, are not restricted to dogs, with wolves also showing attachment-like behaviors to their caregivers (Hall et al., 2015; Hansen Wheat, Larsson & Temrin, 2020; c.f., Topál et al., 2005). Hall et al. (2015) reported that wolf puppies showed more proximity seeking, contact, and greeting behaviors to a caregiver than a stranger after a 2 min separation. Adult wolves also showed attachment-like behaviors similar to dogs in a strange situation test (Hansen Wheat, Larsson & Temrin, 2020); in this study wolves showed heightened differential responding between the caregiver and stranger when the researchers assessed how much the animals explored a room in the presence of a stranger or caregiver, and the amount of greeting behavior the animals directed towards a caregiver and stranger. The authors concluded that the ability to form an attachment with a human caregiver preceded domestication.

Beyond assessing whether dogs’ and wolves’ differentially respond to their owner/caregiver compared to a stranger, another way of assessing their relationship with the owner/caregiver is to measure their preference between the owner/caregiver and another preferred stimulus, such as food. Harlow and Zimmermann (1959) pioneered research into animals’ preferences between what they termed “contact comfort”, from soft body contact, *versus* food. This type of research can speak to what stimuli are valuable to the individual as well as what stimuli might be important in attachments formation. Igel and Calvin (1960) conducted a replication of Harlow and Zimmermann’s study with puppies and found similar results to the original primate work: puppies spent more time with a cloth mother, regardless of whether the cloth mother had provided nourishment to the puppies or not. Nevertheless, the effect of food delivery on preference was still evident: puppies that nursed on a wire mother, while spending more time with the cloth mother overall, still spent more time with the wire mother than did puppies that nursed on a cloth mother. In addition, puppies that nursed on a cloth mother spent more time with the cloth mother than did puppies that nursed on the wire mother. That is, while contact comfort was the catalyst for the increased time allocation to the cloth mother, food delivery still influenced time allocation between alternatives; if a cloth mother also provided food, it was an even more preferred stimulus than if it did not provide food.

More recently, adult dogs’ and wolves’ preferences for human contact *versus* food have been investigated in concurrent choice tests, and are another way of assessing the owner/caregiver-canid relationship. We presented adult dogs a choice between a person providing food and a person providing petting (Feuerbacher & Wynne, 2014) across five sessions in which we varied the availability of food. We found effects of dog population (shelter *vs.* owned), familiarity of the person providing petting (owner *vs.* stranger), the testing context (familiar *vs.* unfamiliar), and schedule of food delivery (ranging from continuous reinforcement to extinction). Most owned dogs preferred food over petting, and would often not approach the petting person even when no food was available; this was in contrast to shelter dogs, which tended to switch readily to the person providing petting when food became slightly less available. We demonstrated that testing the owned dogs in an unfamiliar situation with the owner providing petting produced preferences more similar to those observed in shelter dogs. However, one of the limitations of this study was that dogs were not equally deprived of their owners, who were providing petting, and food; the greatest period of deprivation from the owner was 10 min in this study, whereas they had all been deprived of food for at least 1 hr. This inequality in levels of deprivation could have contributed to the dogs engaging more with the food person than they would have if they were equally deprived of food and the owner.

Lazzaroni et al. (2020) compared hand-reared pack-living wolves to hand-reared pack-living dogs in a concurrent choice test in which the animals could choose to interact with a person who had previously provided social interaction or a person who had previously provided food, although they did not provide these interactions during the test. Hand-reared pack living dogs were more likely to approach either person compared to the wolves, but neither group showed a clear preference for one person over the other. However, as Lazzaroni et al. noted, one issue was that the experimenters had been trainers for those dogs and wolves, and thus had provided both food and social interaction to the animals prior to the experiment. As such, the animals’ might not have been able to sufficiently discriminate which consequence they would receive from which experimenter and this could have contributed to the lack of a clear preference. Additionally, the test was run twice with each animal and the experimenters providing the food and social interaction were counterbalanced across these two tests, which revealed that six dogs and three wolves seemed to base their choice on the trainer and not the form of interaction provided.

Beyond the specific limitations of each study noted above, in both studies, the preference assessment for the delivery of food was conflated with the presence of a human that was either delivering the food (Feuerbacher & Wynne, 2014) or had previously been associated with food (Lazzaroni et al., 2020). While that can speak to preferences for humans who provide food, it does not directly answer the question of how humans compare to food in a concurrent choice. Thus, it remains unclear how dogs and wolves would respond in a choice between an owner/caregiver and food, when the food is provided independently from a human. Depending on the individual animal’s motivation to seek human proximity or interaction, they might allocate more or less responding to the food provided by a human. Thus, in the current study we explored the preferences of hand-reared captive wolves and pet dogs when given a concurrent choice between the owner/caregiver or food, delivered independently of a human, when the dogs and wolves were equally deprived of owner/caregiver and food (a minimum of 4 hr) using a concurrent choice paradigm. Each subject participated in four trials; we measured the canids’ first choice in each trial and the time they allocated to each alternative in each trial. We hypothesized that, given the equal deprivation from the owner and food, dogs would show more sociable behaviors to the human, allocating more first choices and a greater proportion of interaction time to the owner than food. Given that wolves generally have shown a lower level of sociability than dogs, we hypothesized they would should lower sociable behavior in our test as well, showing more first choices and a greater proportion of interaction time allocated to food than the caregiver.

## Materials & Methods

### Wolves

#### Subjects

We tested six hand-reared wolves at Wolf Park located in Battle Ground, IN (see Table 1 for canid demographics). We tested the wolves with the caregiver that interacted with them most on a daily basis. All the caregivers had known the wolf since they were puppies, except for Ayla and Kailani, whose caregiver in this experiment had known them since they were 2 years old. The caregiver that participated in this study for each wolf was identified by other staff members as a highly preferred person for that particular wolf. Wolves were typically fed three times a week with approximately 2.25 kg of a cow or deer carcass provided at each feeding.

**Table 1:** Canid demographics. Demographic data of the wolves and dogs. Age is reported in years (y) and months (m). Sex: F is female, M is male, S is spayed, N is neutered. Wolves were listed as unaltered as they all had sex hormone producing gonads, although not all were reproductively intact (*i.e.,* all females had hysterectomies and Renki had a vasectomy). Under breeds the predominant breed is listed based on owner report and an x indicates the dog is a mix.

Name	Breed	Age	Gender
Ayla	Wolf	10y	UF
Dharma	Wolf	4y	UF
Kailani	Wolf	10y	UF
Renki	Wolf	10y	UM
Wolfgang	Wolf	9y	UM
Wotan	Wolf	9y	UM
Abby	Shetland sheepdog	9y	SF
Aegis	Belgian malinois	2y	SF
Aero	German shepherd	9y	NM
Ally	Golden retriever	2y	UF
Danger Mouse	Pomeranian/Pug	7y	SF
Iorek	German shepherd	5y	NM
Linus	Australian shepherd	6y	NM
Ronin	German shepherd	3y	NM
Saint	Border collie x	9m	NM
Stella	Pit bull	8y	SF

#### Setting

**Figure 1:**
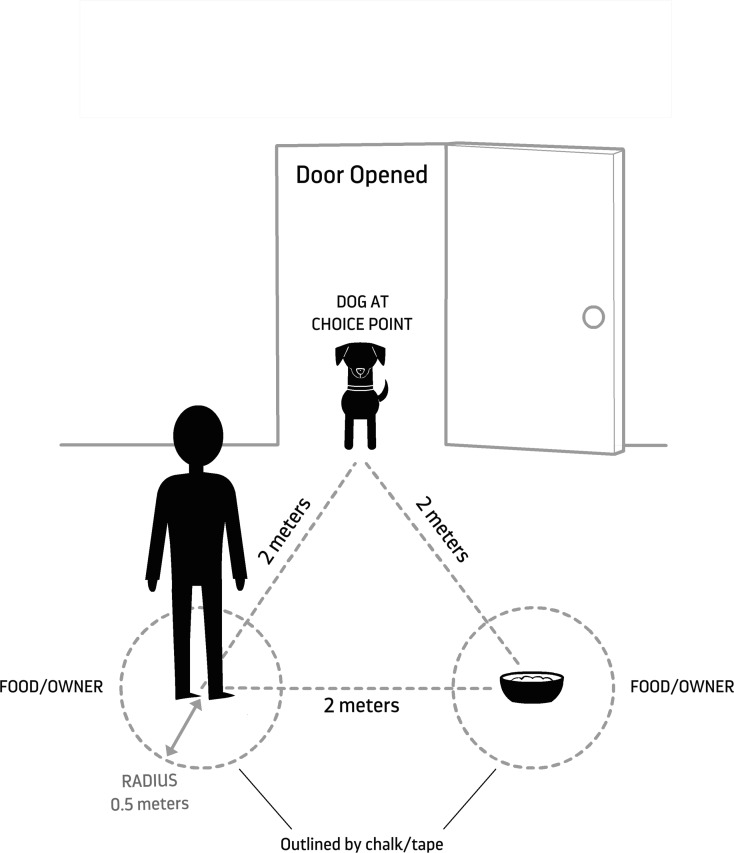
Experimental set up. Schematic and dimensions of the room arrangement for concurrent choice procedures. Created by Lili Chin and used with permission.

One enclosure at Wolf Park was set up for testing according to Fig. 1. The enclosure was familiar to the wolf but was not its home enclosure. The caregiver and food were each stationed in the middle of a circle (0.5 m radius), demarcated by lawn pegs because the testing area was located outside in a grassy area. The two alternatives were arranged 2 m apart (center to center) and equidistant (2 m) from the subject’s point of exit. The angle at which they were placed depended on the expected angle of exit of the subject based on how far the door could swing open. The two alternatives were set up so that they were equally visible as the subject crossed the threshold (Fig. 1).

#### Trials

Prior to each trial, wolves had not interacted with their caregiver or eaten for four hours. Before the wolf was brought to the testing area, the caregiver placed approximately 2 cups of summer sausage onto the ground in one of the circles and stood in the other circle. Summer sausage is a food used to work with the wolves when Wolf Park hosted twice-monthly seminars in spring and fall (R Talbot, pers. comm., July 6, 2020). Thus, it is a familiar food to the wolves although they did not receive it regularly; we chose to present it on the ground rather than a bowl since they were typically fed on the ground, and we wanted to reduce the possibility that wolves would show neophobic behavior to the bowls. The experimenter stood outside of the enclosure to video record and time the trial.

A second caregiver led the wolf from their enclosure to the testing area but did not interact with the wolf other than to lead it to the new enclosure. The caregiver was instructed not to move, encourage, call or make eye contact with the wolf unless it had at least two paws in or on the perimeter surrounding the caregiver. If the wolf did have two paws in or on the caregiver’s circle they were considered “in proximity” to the caregiver and the caregiver verbally praised and petted the wolf. The caregiver interacted in a way that was typical for how they would typically physically interact with the wolf. Outside of the experiment, wolves would solicit physical interaction regularly from caregivers (R Talbot, pers. comm., October 8, 2021); thus, the petting was a typical and valuable activity for the wolves. If the wolf left the caregiver’s circle, the caregiver returned back to being passive. If the wolf re-entered the caregiver’s circle after leaving, the caregiver again verbally praised and petted the wolf.

If the wolf had two paws in or on the circle of the food, or if it was actively eating the food, it was considered in proximity to the food. The wolf was free to alternate between the food and caregiver. The trial ended after 1 min elapsed from the time the wolf crossed the threshold into the enclosure. After the trial, if the wolf had not approached the food, the caregiver showed the food to the wolf, but did not allow it to eat it. This only occurred four times (once for four individual wolves).

Wolves were tested for four trials. Two trials were run per day. One was conducted approximately four hours after any morning medications were dispensed; the second was conducted approximately four hours after the first trial. Wolves had been fed two days before Trials 1 and 2 occurred, as well as after Trial 2 (the day before Trials 3 and 4). The circle in which the caregiver stood varied across trials such that each wolf had two trials with the caregiver on the left and two trials with the caregiver on the right. The sequence of alternation varied randomly across wolves.

### Dogs

#### Subjects

We tested 10 owned dogs. All dogs were at least six months old and had lived with their owner for at least six months (see Table 1).

#### Setting

Dogs were all tested at their own homes. The experimental room was a room adjacent to where the dog was left during the owner’s absence and into which it could safely enter and explore off leash. For most dogs (Aegis, Aero, Danger Mouse, Iorek, Linus, and Ronin), the dogs were released into the garage. The other four dogs were released into a room in the house (kitchen or living room). The owner and food bowl were each stationed in the middle of a circle (0.5 m radius), delineated by duct tape or chalk on the floor (Fig. 1). Aside from being indoors, the experimental set up was identical to that used for wolves, except when space was limited, in which case the circles were placed at slightly shorter distances from the door and with marginally smaller diameters.

#### Trials

Owners left the dog alone for a minimum of four hours before each trial. Time alone ranged from 4 hr to 6 hr, with the majority between 4–5 hr. The only exception was Ronin; he was left alone for 8 hr due to the owner’s work schedule. Dogs had not eaten since the owner left. Before the owner left the house, they prepared their dog’s typical food ration in a dog bowl. Dogs were given their typical brand, type, and the amount of food they would normally be fed as a meal on a daily basis. Upon his or her return, the owner placed the bowl of food in one circle and then stood in the other circle. Owners then faced the door through which the dog would enter the experimental room. Dogs were loose in an adjacent room in which they had been left prior to owner departure. To allow the dog to exit that room and enter the room where it could interact with its owner or food, a second person then opened the door or the owner pulled a string that was tied to the door. The only variation to this was Saint, who was left in his crate when the owner was absent. For Saint, the experimenter went into the smaller room and closed the door. She then let the dog out of the crate and did not interact or make eye contact with it as she let the dog into the testing room and closed the door. Trials lasted for 1 min and started when the dog crossed the threshold into the room where the owner and food were located.

The procedure was identical to that used for wolves except that only one trial was run per day. Dogs were tested for four trials, except for Aegis (tested for six trials) and Iorek (tested for five trials). However, we only analyzed the first four trials from Aegis and Iorek. The circle in which the owner stood varied across trials such that each dog had at least two trials with the owner on the left and two trials with the owner on the right. The sequence of alternation varied randomly across dogs. As with the wolves, if the dog did not approach the food, the owner showed the dog the food but did not allow the dog to eat. This occurred six times (once each for two dogs, and in all four trials for a third dog).

### Analysis

We video recorded all trials and coded from video using Behavioral Observation Research Interactive Software (BORIS; Friard & Gamba, 2016). We coded the dogs’ and wolves’ initial choice (the alternative they came into proximity to first), time spent with owner/caregiver, time spent with food, and whether they consumed the food. While petting is a continuous stimulus, food could be a more discrete stimulus with limited time that dogs and wolves could interact with it. However, we provided dogs and wolves an amount of food that we suspected would allow them to engage with food for the full trial time, and, thus, we would be able to compare time allocated to each alternative between owner/caregiver interaction and food. We recorded if the wolf or dog consumed the food and if they had food available throughout the trial.

A second, independent observer double-coded 25% of videos for interobserver agreement (IOA). We calculated IOA for first choice (number of agreements/total number of trials * 100) and was 100%. We also calculated IOA for time spent with each alternative. If the total time engaged with the alternative from observer 1 was within 1 s of that determined by observer 2, we considered this 100% agreement. If the two times differed by more than 1 s, we calculated percent agreement by total time engaged with the alternative/total time engaged with alternative * 100). Mean IOA for time spent engaged with owner/caregiver was 97% and mean IOA for time spent engaged with food was 96%.

### Ethical statement

The University of Florida Institutional Animal Care and Use Committee (IACUC: 201308062) and Virginia Tech Institutional Animal Care and Use Committee (IACUC: 18-218) approved procedures carried out within this study.

## Results

All six wolves and 10 dogs experienced four trials of the alternatives and all subjects allocated responding to at least one of the alternatives in every trial. However, the video for Linus’ fourth trial and Stella’s third trial were corrupted; thus, Linus’ results are based on Trials 1-3, and Stella’s on Trials 1, 2, and 4. We did record the first choice that Stella made, so the data for first choice for her are based on all four trials.

### Initial preference

**Figure 2:**
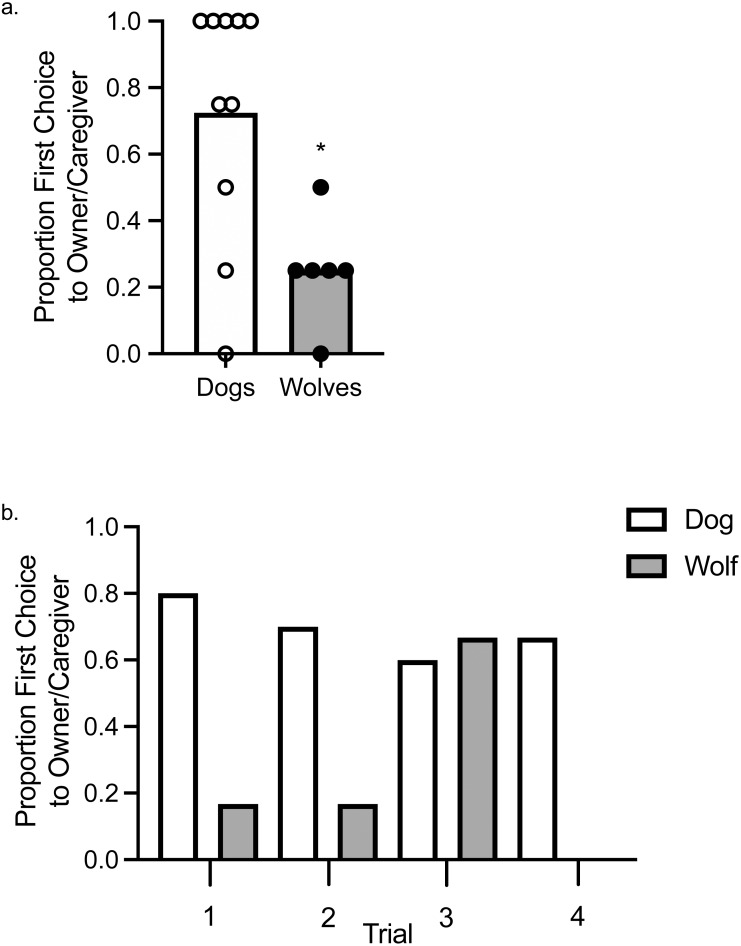
First Choice to owner/caregiver or food. (A) Mean proportion of first choices (bars) allocated to the owner/caregiver in dogs and wolves. Individual data are plotted in the data points. An asterisk (*) indicates *p* < .05. (B) Proportion of first choices allocated to the owner/caregiver in dogs and wolves across individual trials.

Using a one-sample *t-* test to assess whether wolves and dogs chose their owner/caregiver above or below chance levels, we found that wolves chose their caregiver significantly below chance levels, *t* (1, 5) = −3.87, *p* = .012, *d* = 0.16, whereas dogs did not choose their caregiver significantly above or below chance levels, *t* (1, 9) = 1.89, *p* = .091, *d* = 0.36 (Fig. 2A). To assess variation in the first choice response, we fit a Bayesian probit regression model with random effects per animal, controlling for canid type (wolf *vs.* dog), sex, and trial number. Assumptions to fit this model include: observations between animals were assumed independent, whereas observations within an animal were not; we assumed all coefficients were independent *a priori* with normal distributions centered at zero. The variance of each coefficient was learned from the data with inverse-Gamma specifications made *a priori*. Using a 95% credible interval (CI), there was a tendency for canid type to explain the variation observed in the first choice made to food or to the owner/caregiver (E[*β*—Data] = $\hat {\beta }=1.02$, SD[*β*—Data] = 0.52, 95% CI [0.02–2.06]), as well as Trial 3 ($\hat {\beta }$ = −0.97, SD[*β*—Data] = 0.48, 95% CI [−1.94 to −0.07]; Fig. 2B). The trials, other than Trial 3, and sex did not explain the observed variation.

When we included age as a random effect, the overlap between type and age of the animals resulted in canid type no longer explaining the observed variation (E[*β*—Data] = $\hat {\beta }=0.73$, SD[*β*—Data] = 0.58, 95% CI [−0.39–1.90]), nor did age explain the observed variation ($\hat {\beta }=0.34$, SD[*β*—Data] = 0.35, 95% CI [−0.36–1.02]). Age also did not explain the observed variation in an analysis in which we included age but excluded canid type ($\hat {\beta }=0.56$, SD[*β*—Data] = 0.31, 95% CI [−0.04–1.17]). Sex did not account for any variation in either of these models, and Trial 3 only explained the variation in the analysis in which canid type and age were both included ($\hat {\beta }$ = −0.90, SD[*β*—Data] = 0.46, 95% CI [−1.80 to −0.02]), and not in the analysis that only included age.

### Time allocation

To ascertain whether we had provided sufficient food such that both interaction with the owner/caregiver and food were continuous stimuli, we assessed whether there were any trials in which the animal had consumed the food before the trial was over. This occurred only once for wolves (Trial 2 for Kailani, when she finished consuming the food 7 s prior to the end of the session); and twice for dogs (Trials 2 and 3 for Alley, when she finished consuming the food 10 s and 7 s prior to the end of the session). In all other trials, food was still available to the animal at the end of the trial. Thus, we deemed comparing time allocated between alternatives reasonable as both were available for 99% of total trial time.

**Figure 3:**
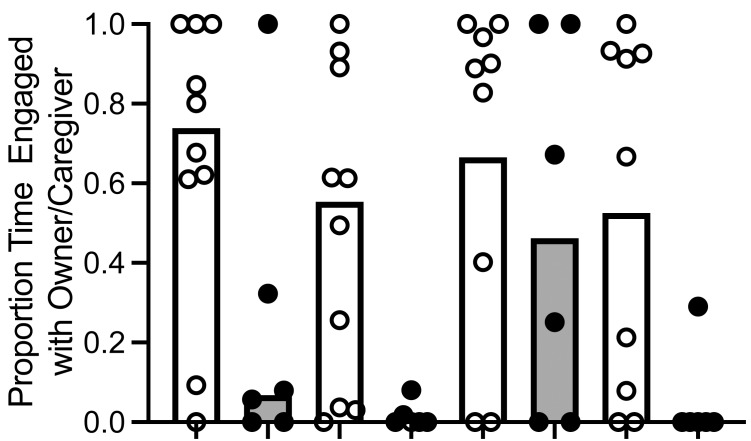
Proportion of time allocated to owner/caregiver or food. Median proportion of time allocated to owner/caregiver or food in dogs and wolves across trials (bars). Individual data are plotted in the data points. We found a main effect of canid type: dogs spent a significantly greater proportion of time with the owner than wolves spent with the caregiver, *F* (1, 12) = 5.737, *p* = .039, *η p*^2^ = .31.

To determine if dogs and wolves differed in the proportion of time they allocated to each alternative, we used an aligned rank transformation (Kay & Wobbrock, 2019; Wobbrock et al., 2011) in R Studio and employed a linear model to analyze the transformed data. For ease of interpretation, we have reported results in untransformed medians and IQR. We found a main effect of canid type: dogs spent a significantly greater proportion of time with the owner (median proportion = .66, IQR [.32, .87]) compared to wolves (median proportion = .22, IQR [.10, .31]; *F* (1, 12) = 5.737, *p* = .039, *η*p ^2^ = .31; Fig. 3). We also found a main effect of trial, *F* (3, 40) = 3.77, *p* = .018, *η*p ^2^ = .22). When we conducted post hoc Wilcoxon signed-rank tests, dogs and wolves allocated a significantly higher proportion of time to the owner/caregiver in Trial 1 (median proportion = .62, IQR [.07, .88]) compared to Trial 2 (median proportion = .14, IQR [.0, .61]; *Z* = −2.27, *p* = .023, *r* = .53), and Trial 3 (median proportion = .82, IQR [.12, .98]) compared to Trial 2 (*Z* = 2.12, *p* = .034, *r* = .57), although these effects disappeared when we conducted a Bonferroni correction, resulting in *α* = .008. Furthermore the interaction between canid type and trial was not significant *F* (3, 40) = 1.24, *p* = .309, *η*p ^2^ = .08).

While we did not conduct a statistical analysis on time allocated to each alternative x canid type x trial due to the small sample size and increased likelihood of an underpowered analysis, we did calculate the time allocated to each alternative: time allocated to owner/caregiver (median of individual dog means = 18.08, IQR [8.51, 27.32]; median of individual wolf means = 1.88, IQR [1.13, 2.69] and time allocated to food (median of individual dog means = 12.06, IQR [3.23, 31.5]; median of individual wolf means = 16.66, IQR [6.15, 25.53]).

We also quantified the pattern of interaction with food. In four trials (10.5% of trials; one trial each for four individual dogs), the dog approached the food and did not eat it on the first approach, but did consume it on a subsequent approach. In five trials (20.8%; four trials for one wolf and one trial for a second wolf), wolves did the same. Additionally, in 12 trials (31.5% of trials), six individual dogs approached food but did not eat it during the trial; in nine trials (37.5%), three individual wolves approached food but did not eat it during the trial.

## Discussion

We investigated how wolves and dogs would allocate their first choice and their time between their owner/caregiver or food when deprived equally of both and when the two alternatives were clearly discriminable, with food being delivered independent of a human. These parameters addressed some of the limitations of prior studies (Feuerbacher & Wynne, 2014; Lazzaroni et al., 2020). Dogs did not approach their food or their owner above chance levels, although on average they chose the owner first on 70% of the trials. They also allocated about 50% of their total interaction time to each alternative. Although neither measure showed strong preference for the owner over food, both measures are slightly higher than those observed in Feuerbacher & Wynne (2014), in which dogs were more deprived of food (at least one hour) than the owner (several minutes) and food was delivered by a person.

In Feuerbacher & Wynne (2014), the Owner-Familiar is the most similar condition to our current study, as, in that condition, the owners provided petting in a location familiar to the dog, while a stranger provided food. Using the first session as the most similar comparison (the dog was more deprived of the owner compared to subsequent sessions and food was continuously available), dogs chose the owner first in slightly less than 60% of the trials compared to the 70% we observed in the current study. In the earlier study, dogs also allocated less than 10% of their responding in the session to the owner (Feuerbacher & Wynne, 2014), whereas they allocated approximately 50% of total interaction time to the owner in the current study. However, the sessions in Feuerbacher & Wynne (2014) were longer (5 min compared to 1 min in the current study) and the data we are using in the current study was generated by testing dogs on repeated, identical trials. Nonetheless, because we had access to the raw data from Feuerbacher & Wynne (2014), we calculated the percentage of time dogs allocated to the owner during only the first 1 min of the session to make it more comparable to the current study. We found that when only looking at the first minute of the trial, dogs allocated 44% of their interaction time to the owner. This was only slightly lower than the 50% we observed in the current study and much more similar than the results from the full 5 min session. This difference also suggests that dogs might show more responding to their owners in the first 1 min of a session, but might allocate more responding to the food alternative when given longer sessions.

The results from our current study suggest that even with using much greater deprivation from the owner in the current study, we still only saw minimal shifts in preference compared to earlier studies. It is possible that even a short deprivation from the owner, such as the few minutes that the dogs experienced in our 2014 study (Feuerbacher & Wynne), created similar motivation as did the much longer deprivation (at least 4 h) in the current study. Our results were also similar to those of Lazzaroni et al. (2020), in which they tested hand-reared, pack-living dogs that had been not been fed and had not interacted with either person since the day before, although they did interact briefly (30 s) with each during the pre-test phase that occurred immediately before the test phase, which could have reduced the deprivation they were experiencing. They also found dogs showed no clear preference for either alternative. Further assessing the impact of the owner absence on a dog’s motivation for the owner compared to other stimuli is warranted and could be useful in understanding and treating separation-related behavioral problems in dogs (see De Assis et al., 2020; Feuerbacher & Muir, 2020; Mundell et al., 2020).

Beyond equalizing deprivation, we also removed the human from food delivery so that the choice was more clearly between a human and food. In prior studies, food delivery had been accomplished by one of the assistants giving the dog food, (*e.g.*, Feuerbacher & Wynne, 2014) or testing the dog’s preference for a person who had recently provided the animal food (*e.g.*, Lazzaroni et al., 2020), both of which might conflate the dog’s motivation for human proximity or interaction with their motivation for food. Nevertheless, this change in food delivery did not seem to have a large impact on our data compared to earlier studies in which a human delivered food (Feuerbacher & Wynne, 2014) or in which the choice was between a human who had recently provided social interaction or food (Lazzaroni et al., 2020).

Wolves, on the other hand, showed a statistically significant first choice for food, choosing food in 75% of the trials. Additionally, they also allocated on average over 80% of their total interaction time to food. Lazzaroni et al. (2020) investigated wolves’ first choice and time allocated between a person who had recently been associated with providing social interaction and a person who had recently been associated with providing food. While Lazzaroni found tendencies that parallel our results, with wolves more likely to approach the person who had provided food than social interaction, and spending longer with the person providing food, neither of these results were significantly different from the similarly-reared dogs they tested. As the authors noted, however, both humans in their study were trainers for the wolves and had provided both food and social interaction to them; thus, this lack of preference might be reflective of a lack of discriminability between the options. In our study, our two alternatives were readily discriminable and we saw wolves show a preference for food in both measures.

We found that wolves differed from dogs in their behavior in a concurrent choice between the owner/caregiver and food. In terms of first choice, wolves allocated their first choice to the food at above chance levels, while dogs distributed their initial choices more equally between the owner/caregiver and food. In our Bayesian probit regression that included canid type, we found that canid type explained the variation we observed in our first choice data, with dogs choosing the owner first at a higher frequency than wolves across all trials except for Trial 3, which was the other variable that explained the observed variation. In terms of proportion of interaction time allocated to each alternative during each trial, dogs allocated a significantly greater proportion of interaction time to the owner/caregiver compared to food than the wolves did. Of course, proportions could be driven by very different magnitudes of how time was allocated to the alternatives. However, the magnitudes of time spent with food were similar and dogs spent an absolute greater amount of time with the owner/caregiver than wolves. That is, the difference in proportional time allocated seems to be driven mainly by time allocated to the owner/caregiver; when comparing the individual animal means, dogs spent a median of 16 s more with the owner/caregiver than wolves did, and wolves only spent a median of 4 s more with the food than dogs did.

The differences we detected between dogs and wolves in the first choice data and proportion of interaction time data align with what we might predict based on prior comparisons between wolf and dog behavior in which dogs have shown greater sociability with humans than wolves (*e.g.*, Bentosela et al., 2016; Lazzaroni et al., 2020; vonHoldt et al., 2017), including when comparing hand-reared, pack-living dogs’ with hand-reared, pack-living wolves (Lazzaroni et al., 2020). Nevertheless, the multifactorial nature of behavior and the differences in rearing and current conditions of the dogs and wolves we tested preclude us from making firm conclusions about the source of this behavioral difference. We discuss below a variety of factors that could be potential contributors to the observed behavioral differences between wolves and dogs, beyond genetic differences possibly tied to domestication. All are sources for future research into the impacts of phylogeny, ontogeny, and current conditions on wolf and dog behavior.

First, dogs were fed twice a day, while wolves were fed tri-weekly. The impact of this feeding schedule on motivation, in fact, might account for the difference observed in Trial 3 in the Bayesian probit regression, which appears to be driven largely by a change in the behavior of wolves a majority of first choices to the caregiver. This trial was conducted on the second day of testing. Wolves had been fed their tri-weekly ration after the second trial of the first testing. Thus, although they were as deprived as dogs from any food (after receiving morning medication) during Trial 3, this might have influenced their behavior towards the food alternative in Trial 3 such that the human was now more valuable than the food, and their greater food motivation was possibly evident in their behavior on the other trials. Additionally, wolves received summer sausage less frequently than dogs received their normal rations, which could have also increased the wolves’ motivation for the food alternative. Nevertheless, the magnitude of time allocated to food between dogs and wolves was similar and there was an equal number of trials in which wolves and dogs approached the food but did not eat it. For logistical reasons, we also tested wolves twice a day instead of once. This could have impacted their performance compared to dogs as well, although we only detected a difference in first choice in Trial 3.

The animals’ feeding schedules additionally might have impacted the dogs’ and wolves’ observed preference for the owner or food. While we arranged the experimental conditions so that dogs and wolves were equally deprived of the owner/caregiver and food, which was not controlled for in prior studies (*e.g.*, Feuerbacher & Wynne, 2014), food could still be perceived as more of a limited resource since the dogs and wolves were fed on a schedule (twice per day for dogs; three times per week for wolves), whereas human company was more freely available. The difference in availability of food compared to availability of the owner is especially striking for dogs, which typically have extended access to the owner when the owner was home. None of the dogs tested were free-fed; it is possible that if we tested free-fed dogs, their allocation to the owner/caregiver could have been greater than what we observed in the dogs we tested, for which food was still potentially a more limited resource. Further, it is possible that even the same duration of deprivation from the owner/caregiver and food could differentially affect the value of each stimulus. Parametric analysis of deprivation of both resources could help elucidate the effects on preference in both species, as could testing animals with different feeding schedules.

Second, the dogs and wolves we tested likely differed in their day-to-day interactions with humans, even though the wolves were similar to dogs in that they were socialized with humans (hand-reared) and had regular interaction and training with their caregivers. For example, as they still lived in outdoor enclosures, rather than as house companions, the absence of the caregiver for 4 hrs might not have produced the same motivation in the wolves as the absence of the owner would in the tested dogs, which lived in the home with the owner. The regular interaction with the owner might make the owner’s absence more salient to the dogs than the caregiver’s absence for wolves; on the other hand, the more regular human interaction that dogs receive might make human interaction less valuable to dogs than to wolves. Nevertheless, our results are still similar to results from comparisons of dogs and wolves that were reared and managed equivalently at the Wolf Science Center (Lazarroni et al., 2020).

Third, while we also attempted to equalize the relationship between the owners and caregivers by asking the person who was reported to have the closest relationship with the animal to participate, it is possible that the two populations could have differed in the relationship they had with the owner/caregivers, beyond any phylogenetic differences in attachment (*e.g.*, Gácsi et al., 2005). Differences in the relationship could translate to how preferred the person is compared to other stimuli, such as food, or the impact deprivation from the owner/caregiver has on the animal. Of the six wolves tested, four knew the caregiver in this study since they were puppies; the two wolves that were tested with the caregiver that knew them starting when they were two years old, however, did not show responding either in the first choice or the proportion of time allocated that stood out as outliers. Of course, since we did not quantify the relationship between dogs and their owners, the same factors that might impact differences between dogs and wolves could generate some of the individual variation we observed in dogs, with owners differing in value based on the dog-owner relationship. Quantifying the wolf-caregiver and dog-owner relationship and evaluating whether it correlates with the performance on concurrent choice tasks would be fascinating.

We also attempted to equate our experimental conditions across dogs and wolves by testing animals in areas with which they were familiar; for six dogs and all six wolves, they were tested in areas familiar to them but that they did not spend the majority of their time (*i.e.,* garage for dogs). For four dogs, however, housing constraints were such that they were tested in areas where they spent more time (main rooms of the house). We did not observe that these four dogs were outliers in any way, but this difference should be noted. Dogs were also tested indoors while wolves tested outdoors, but, as noted, the spaces into which they were released were of similar familiarity. Nonetheless additional environmental stimuli such as odors might have differentially affected animals tested outdoors compared to indoors and future studies should seek to investigate the impact of these conditions.

Additionally, the impact of age on sociability and preference warrants further investigation. In our Bayesian probit regression, the addition of age removed canid type as a factor explaining the observed variation. This is because age correlated with canid type in that five wolves were older than eight of the dogs, which limits to some extent what we can learn from either variable. For example, when both age and canid type were included in the probit regression model, both variables appear un-important (the CIs overlap zero). However, when only canid type is included, we do find that canid type explains the observed variation at the 95% CI. Still, when only age is included, age does not explain the observed variation at either the 95% or 90% CI. Thus, while canid type by itself does explain the observed variation and age by itself does not, the amount of overlap between the two variables indicates a need for further investigation. To more fully discern the influence of age on animal choice, conditional on type, more data are needed.

In a concurrent choice between a cloth mother and a wire mother, puppies allocated a significantly greater amount of time to the cloth mother, even among puppies fed from the wire mother (Igel & Calvin, 1960). This is at odds with what we observed in adult dogs. Whether this difference from our results is due to different experimental procedures or an effect of age cannot be determined. Studies specifically investigating the role of age on sociability and preferences for social interaction *versus* other stimuli, such as food, would be incredibly valuable.

Finally, our sample size, especially for wolves, was small and limits the strength of our conclusions; future research should continue to explore these topics with greater numbers of animals. Nevertheless, we observed substantial individual differences in behavior, especially in dogs. Some dogs exclusively allocated time to the owner, while others exclusively allocated time to food. Wolves, on the other hand, showed much less variation in both measures. While an interesting difference, our study was not designed to elucidate whether that difference is a reflection of species differences, or a product of the dogs having a greater genetic variation or greater variation in past and current rearing conditions compared to the wolves we tested.

Still, within individual animals we often saw consistencies in responding: across the four trials, 10 animals (five wolves and five dogs) each allocated a similar amount of time to the owner/caregiver (10 s range within each individual), and seven animals (two wolves and five dogs) allocated a similar amount of time to food (10 s range within each individual). This suggests that, within individuals, our trials produced similar motivational states within that individual. Nevertheless, we did not explore the variables that influenced the differences between individuals, which could include individual differences in sensitivity to deprivation to either the caregiver/owner or food, past learning histories, genetic or epigenetic differences in genes associated with social behavior or food hunger, or a confluence of several of these. We hope that future research will explicitly investigate such variables.

## Conclusions

We investigated how wolves and dogs would allocate their responding between their owner/caregiver and food, under conditions of equal deprivation and when the food was not delivered by a human. We found little evidence that the additional deprivation from the owner changed the dogs’ responding substantially and, overall, dogs did not show a preference for the owner or food. However, there was substantial individual variation between dogs with some exclusively allocating responding to the owner and others exclusively allocating responding to food, suggesting we should explore the possible factors determining these differences in future studies. On the other hand, wolves showed a preference for food in both the first choice and proportion of time allocated, with less individual variation than we saw in dogs. While our results are in line with dogs being more sociable than wolves (Lazzaroni et al., 2020; vonHoldt et al., 2017), we have noted several ontogenetic factors and population differences between the dogs and wolves we compared, which could account for the observed differences. Further investigation into how different housing and feeding practices impact wolf and dog behavior are warranted. More clearly understanding the phylogenetic and ontogenetic factors, and their interactions, that influence these observed differences can enhance our understanding of dog behavior and the human-dog relationship.

## Additional Information and Declarations

### Data Availability

The following information was supplied regarding data availability: Data is available at VTechData: Erica N Feuerbacher; Lindsay Taylor Isernia; Clive D. L. Wynne (2020): Dogs’ and wolves’ differ in the time spent with an owner/caregiver or food in a concurrent choice procedure. University Libraries, Virginia Tech. Dataset. https://doi.org/10.7294/xdx9-se32.
